# The multi-modal digital heart: From future perspective to challenges

**DOI:** 10.1515/jtim-2026-0052

**Published:** 2026-06-13

**Authors:** Xinyue Zhao, Kai Wang, Ming Xu

**Affiliations:** Department of Physiology and Pathophysiology, School of Basic Medical Sciences, State Key Laboratory of Vascular Homeostasis and Remodeling, Beijing Advanced Center of Cellular Homeostasis and Aging-Related Diseases, Peking University, Beijing, China; Department of Cardiology and Institute of Vascular Medicine, Peking University Third Hospital, State Key Laboratory of Vascular Homeostasis and Remodeling, NHC Key Laboratory of Cardiovascular Molecular Biology and Regulatory Peptides, Peking University, Beijing, China

## Introduction

Heart diseases are the most lethal disorders and their complex mechanisms remain elusive. To address this, the digital heart, an in-silico model, representing gene expression, anatomy, or function of cardiac system in healthy individuals or patients, has emerged. By integrating multi-modal information such as transcriptomics, proteomics, clinical imaging, and electrophysiology data, a digital cardiac model can be established. The digital heart can facilitate the investigation of the mechanisms of cardiac diseases. Moreover, it can also be applied in precision medicine, *i.e*. the digital twin, for phenotyping, diagnosis, risk stratification, and therapeutic prediction.^[1]^

There are other digital organs (brain, lung, liver). The digital heart is distinguished from them by its intense multi-physics integration. The native four-chamber structure emphasizes the significance of blood-flow simulation. The electromechanical pump function requires the integration of multi-scale signals (milliseconds for changes in ion channels and action potentials, seconds for activities in the organ rhythm level, and minutes for regulation of myocardial contractile function and hemodynamic feedback). Fortunately, these electromechanical processes are monitored by mature techniques such as high-resolution magnetic resonance imaging (MRI), electrical mapping *via* electrocardiogram (ECG) with quantitative, time-resolved data unmatched by other organs.

Overall, this perspective discusses about digital heart fusing information about transcriptomics, spatial proteomics, clinical imaging, and electrophysiology. Furthermore, heart organoids can be a valuable data source for digital heart modeling as well as a platform to test the accuracy of the model. The challenge, however, may be the development of a deep learning tool capable of processing these vast and complex datasets.

## Transcriptomics in digital heart

Single-cell RNA sequencing (scRNA-seq) profiles heterogeneous cardiac cell clusters but loses spatial context. Spatial transcriptomics platforms like scStereo-seq bridge this gap. Integrating these methods can construct of a single-cell-resolution 3D atlas of human heart to identify cell types, detect spatially molecular signatures, monitor ion channel activities underlying electrophysiological signals, and analyze cell-cell interactions and spatial proximities. Kanemaru’s article combines scRNA-seq and spatial transcriptomes to profile human heart cell clusters, discover unbiased niches or microenvironments, evaluate ion channel genes expression and find drugs targeting pacemaker cells of cardiac tissues.^[2]^ Together, these data provide significant transcriptomics patterns of the organization and function of human heart.

## Spatial proteomics in digital heart

Spatial proteomics techniques, such as Co-Detection by Indexing (CODEX), portray hundreds of markers *in situ*, reveal the protein expression level of genes identified through scRNA-seq, and confirm the molecular characteristics of cell groups. Although performing CODEX technology in human heart area is a frontier and so far no such studies have been reported, CODEX has been used in studies of tumor niches.^[3]^ By combining spatial transcriptomics, single-cell nucleus sequencing and matched CODEX data, Mo examined discrete cancer cell types segregated by stromal components, *i.e*. “tumor microregions”, within spatial contexts. The authors combined CODEX data with spatial transcriptomics and utilized a multimodal 3D reconstruction tool to confirm the tumor–immune interphase niches and interactions. Looking ahead, these advances are likely to be applied in cardiovascular studies. Applying CODEX to heart can map ion channels, extracellular matrix proteins, and signaling molecules informative for electrophysiology or mechanobiology simulations. However, due to autofluorescence caused by high mitochondrial content in muscle cells and collagen-and elastin-rich connective tissue, the signal-to-noise ratio is low, affecting accurate protein quantification. Dense and highly packed cardiomyocytes and abundant extracellular matrix can hinder antibody diffusion, causing incomplete staining and biased protein detection. In summary, spatial proteomics creates cell atlas in protein level, and confirms cellular identity and gene expression within spatial contexts.

**Figure 1:**
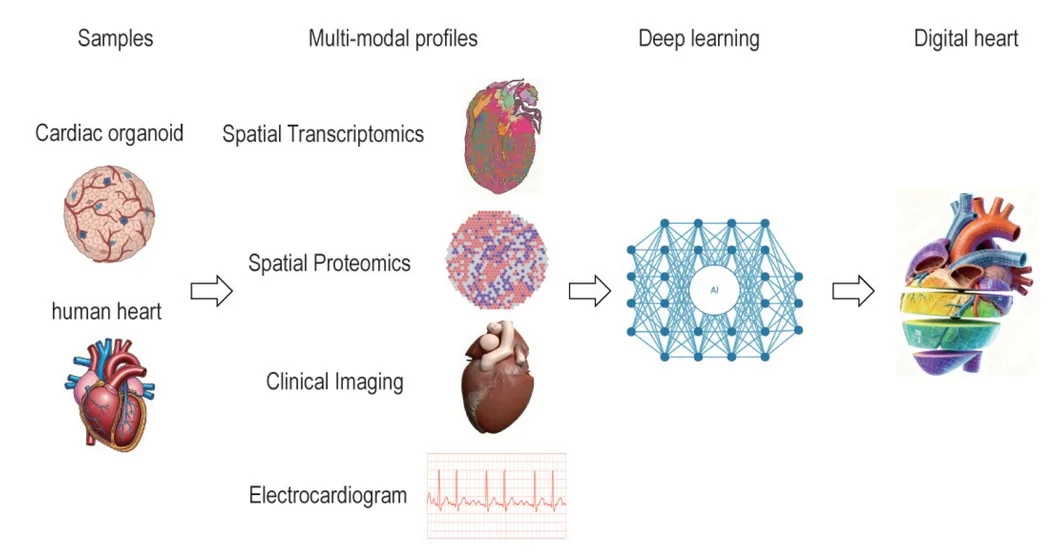
Schematic of the digital heart construction pipeline. Cardiac organoids or human hearts are analyzed for spatial transcriptomics, spatial proteomics, clinical imaging and electrocardiogram. These data are then integrated and processed by a deep learning tool to construct a digital heart model. Icons in the picture are obtained from BioRender and the picture is produced using Adobe Illustrator. Created in BioRender. Wang, K. (2026) https://BioRender.com/ix8b1g2.

## Clinical imaging in digital heart

Clinical imaging *via* computed tomography (CT) or MRI noninvasively visualizes cardiac anatomy and function (ventricular volumes, ejection fraction). Strocchi built 3D four-chamber heart models from CT images of twenty-four patients with heart failure, simulate electro-mechanics and ejection fraction.^[4]^ Zou performed a 3D reconstruction of cardiac and pulmonary structures with MRI data based on spatial transformer networks, identifying the key cardiovascular structures and capturing different phases of the cardiac cycle with enhanced image quality, improved motion robustness, and efficient memory usage.^[5]^ For surgical planning, Lipper generated digital twins from MRI and/or CT images, altering surgical plans for patients with complex heart defects in over half of the cases, increasing patients’ benefits and preventing negative post-surgery effects.^[6]^ Arevalo generated a personalized cardiac model based on MRI data from post-infarction patients to predict the risk of arrhythmia development. Its performance emphasizes the superiority of digital cardiac models in prognosis-related applications.^[7]^ Collectively, CT and MRI facilitate digital whole-heart modeling by providing high-resolution anatomy, quantifying functional parameters, mapping pathological features such as scars or fibrosis, and supporting electro-mechanics simulations.

## Electrophysiology in digital heart

ECG provides high-temporal-resolution data on cardiac electrical activity (depolarization, repolarization, rhythm, conduction). ECG waveforms are integrated with imaging data and serve as a validation benchmark for simulated activity. Camps integrated ECG information with MRI data, extracted personalized activation and repolarization characteristics, and generated digital twins, enabling in-silico ECG simulations such as QT intervals (QTI) and T peak to T end intervals (TTI) under different drug dosages or in the absence of drugs, supporting the evaluation of personalized drug responses.^[8]^ As the dose of Ikr blocker Dofetilide increases, the durations of simulated QTIs and TTIs increase, which matches the clinical trends and demonstrates the capability of personalized digital twins to evaluate the effects of arrhythmic vulnerability to drugs. Similarly, Gillette used twelve standard ECG signals and MRI-derived 3D whole heart models to generate patient-specific cardiac digital twins.^[9]^ The fidelity of computed ECG from the digital twins was verified against the gold standard bidomain model. ECG is thus essential for constructing and validating the electrophysiological fidelity of digital hearts.

## Cardiac organoid and digital heart

Cardiac organoids are 3D stem cell-derived models that recapitulate cardiovascular architecture and function. They serve a dual role: as a vital data source for digital heart models, providing transcriptomic, proteomic, mechanical, and electrophysiological data from patient-specific induced Pluripotent Stem Cells (iPSCs), and as an experimental platform to validate model predictions and test drug responses.^[10]^ The feedback on drug toxicity and efficacy can be used as input to further improve the digital heart model.

## Difficulties and challenges

Building a representative digital heart requires computational tools to integrate large-scale, multi-modal data. Generative deep learning models (autoencoders, Generative Adversarial Networks [GANs]) are promising for handling high-dimensional data and synthesizing integrated representations. Various tools integrate ECG and MRI data and generate cardiac digital twins.^[8,9]^ A versatile tool managing all data modalities (transcriptomics, proteomics, imaging, electrophysiology) with capabilities for image processing and 3D stitching is lacking. Data standardization is another challenge. Consistent acquisition protocols and quality control across centers are essential. The lack of semantic interoperability further impedes multi-omics data integration. Computational cost is significant as processing high-dimensional omics data or running complex simulations requires substantial resources. Ethical issues regarding data privacy and data regulation should also be addressed such as the acquisition, storage, and training of sensitive health data. The re-identification of patients’ private information should be avoided and the model should be tested using differential privacy measures. For example, the generative model should be evaluated under membership inference attacks.^[1]^ Data perturbation and tokenization reduce the risk of training data leakage. Moreover, digital cardiac model may be considered a medical device, raising requirements for validation, regulation, and oversight.

## Perspective

Digital heart, which integrates spatial transcriptomics, spatial proteomics, clinical imaging, and electrophysiological information, will become a powerful tool for study of the cardiac system, and heart organoid will be a significant data source to enhance digital heart models. However, corresponding cross-modal deep learning methods need to be developed in the future.
